# Selection of Soil- and Wastewater-Derived Indigenous Anaerobic Bacterial Isolates for Enhanced Lignocellulosic Substrate Degradation and Methane Production

**DOI:** 10.3390/microorganisms14030530

**Published:** 2026-02-25

**Authors:** Katerina Klavdianou, Georgios Manthos, Dimitris Zagklis, Sameh S. Ali, Michael Kornaros

**Affiliations:** 1 Laboratory of Biochemical Engineering & Environmental Technology (LBEET), Department of Chemical Engineering, University of Patras, 26504 Patras, Greece; klavdianoukaterina2000@gmail.com; 2 Department of Environmental and Resource Engineering, Quantitative Sustainability Assessment Section, Technical University of Denmark, Bygningstorvet, Building 115, DK-2800 Kongens Lyngby, Denmark; gema@dtu.dk; 3 Department of Industrial Engineering and Management, International Hellenic University (IHU), 57400 Thessaloniki, Greece; zagklis@ihu.gr; 4 Botany and Microbiology Department, Faculty of Science, Tanta University, Tanta 31527, Egypt; samh_samir@science.tanta.edu.eg

**Keywords:** anaerobic digestion, bioaugmentation, BMP, enzymes, kraft lignin, lignin degradation, *p*-coumaric acid

## Abstract

Lignocellulosic biomass is an abundant renewable resource, yet its effective utilization remains limited due to its structural recalcitrance, primarily attributed to lignin. While aerobic lignin-degrading microorganisms, particularly fungi, have been extensively studied, much less is known about bacteria capable of lignin depolymerization under low-oxygen conditions. This study focused on the isolation and evaluation of native anaerobic bacterial cultures capable of degrading lignin-derived compounds to enhance biogas production. Soil samples from decaying vegetation and olive mill wastewater were used as microbial sources. Enriched cultures were developed anaerobically using kraft lignin and *p*-coumaric acid as sole carbon sources. Twelve pure bacterial strains were isolated and screened for their ligninolytic activity. All strains were able to degrade *p*-coumaric, with the highest biomass concentration reaching 387 mg L^−1^ and maximum substrate consumption rate at 438 mg L^−1^ d^−1^. When kraft lignin was used as sole carbon source, 9 out of 12 strains showed growth, with a maximum of 55 mg L^−1^ over 11 days. Enzyme activity assays confirmed the production of lignin peroxidase and laccase, with highest values at 2.10 and 0.15 U mL^−1^, respectively, even under conditions of limited oxygen. The enriched cultures were applied in biomethane potential (BMP) batch tests, resulting in increased methane production. The best performing culture resulted in a bioaugmentation percentage of 174% compared with control. These findings suggest that native ligninolytic bacteria can serve as promising bioaugmentation agents in anaerobic digestion of lignocellulosic waste.

## 1. Introduction

Lignocellulosic biomass (LB) represents the most abundant form of hard-to-degrade biomass globally, reaching 181.5 billion tons annually [1], consisting mainly of forest and agricultural residues, such as wood from deciduous and coniferous forests, municipal solid waste, and paper industry waste [2,3]. The three main components of LB are cellulose (40–60% *w*/*w*), hemicellulose (20–35% *w*/*w*) and lignin (15–45% *w*/*w*) [4]. While cellulose and hemicellulose can be converted into fermentable sugars for the production of high value bioproducts, the inherent recalcitrance of LB to enzymatic hydrolysis remains a major obstacle for its large-scale industrial use. This resistance can be attributed to structural factors such as crystallinity [5], molecular size [6], and pore size and chemical factors such as degree of polymerization [7], syringyl to guaiacyl ratio (S/G ratio) [8], and hydroxyphenyl (H) content [9], but most notably the chemical linkages between lignin and the two other biopolymers, known as the Lignin Carbohydrate Complex (LCC) [4,10,11].

Both fungi (soft, white and brown-rot fungi [12]) and bacteria (*Firmicutes*, *Proteobacteria*, *Bacteroidetes*, *Actinobacteria*, etc. [13]) are capable of producing lignocellulolytic enzymes, which are categorized into three main groups according to their substrate target: cellulases, hemicellulases and ligninolytic enzymes. These enzymes can also be classified as auxiliary and modifying. The auxiliary enzymes cannot degrade lignin on their own; however, through the sequential action of multiple proteins and the oxidative action of H_2_O_2_, they make lignin hydrolysis feasible. Examples include glyoxal oxidase, aryl-alcohol oxidase, and pyranose 2-oxidase [14]. The main modifying enzymes are laccases and heme peroxidases, the latter including manganese peroxidase (MnP), lignin peroxidase (LiP), and versatile peroxidase (VP) [15].

This study focused on the isolation of microorganisms with lignin-degrading capacity from natural environments, such as soil under different trees, where lignocellulosic biomass gradually decays, as well as wastewaters such as olive mill wastewater (OMW) and anaerobic sludge, which are residues typically having a high lignin content [16].

A sequential methodology was applied: liquid cultures were prepared from every sample source (soil under trees and wastewaters) under anaerobic and mesophilic conditions (37 °C). These mixed cultures were further used as sources for the isolation of morphologically distinct colonies. Both mixed and isolate cultures were initially cultivated on *p*-coumaric acid, a phenolic compound that serves as a structural model for the *p*-hydroxyphenyl (H) units of lignin [17]. Due to its lower molecular complexity compared to the lignin polymer, *p*-coumaric acid allowed for efficient primary screening of ligninolytic potential of the isolates. Subsequently, the cultures were transitioned to kraft lignin, a technical form of lignin, to complete a gradual acclimatization to complex phenolic environments. Finally, the ability of the mixed cultures to enhance methane production was evaluated through biomethane potential (BMP) assay using corn silage as a natural lignocellulosic substrate.

Research on lignin biodegradation have extensively investigated aerobic microorganisms, particularly white-rot fungi, which are known for their efficient oxidative enzyme systems [18]. In contrast, the anaerobic degradation of lignin has been far less explored. Only a limited number of studies have focused on anaerobic bacteria or consortia capable of modifying or partially degrading lignin, and the mechanisms involved remain poorly understood [19]. This knowledge gap highlights the need to identify and characterize new ligninolytic microorganisms that can operate under anaerobic conditions, which are relevant to many natural and engineered environments where oxygen availability is limited.

The findings of this study are valuable for both researchers and practitioners: researchers gain insight into a readily applicable strategy for discovering new ligninolytic bacteria from widely available natural sources, a beneficial approach for potential industrial applications, while practitioners benefit from an easy-to-implement and cost-effective protocol. Overall, this work proposes an integrated and practical methodology to isolate, acclimate, and select the most promising ligninolytic microorganisms for effective use in lignocellulosic biomass valorization and bioenergy production.

## 2. Materials and Methods

### 2.1. Experimental Design

**Figure 1:**
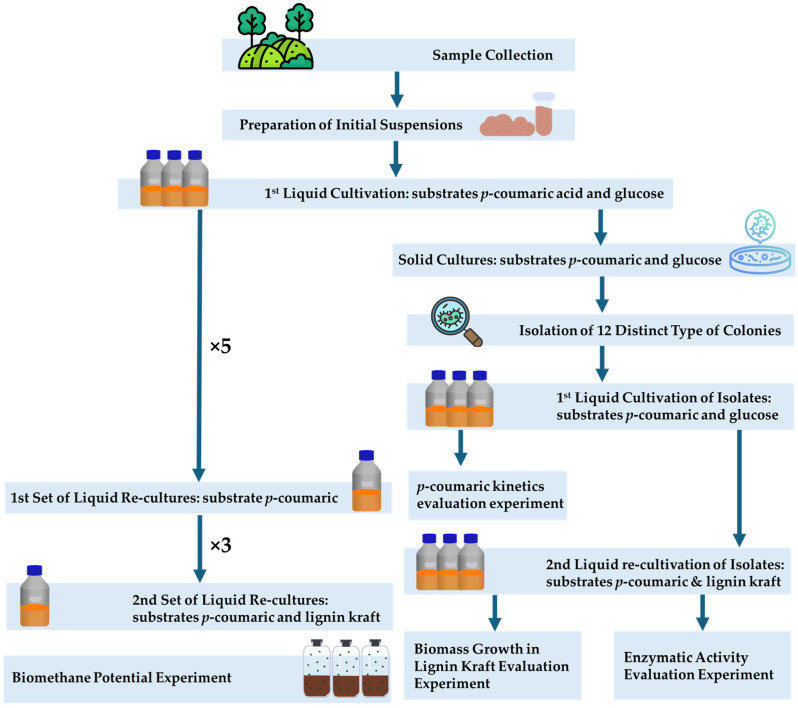
Schematic representation of experimental design.

The overall methodology is summarized in Figure 1, illustrating the full sequence of enrichment, isolation, and evaluation steps used in this study.

After sample collection, the first step was to enhance the growth of anaerobic bacteria by cultivating them in Anaerobic Basic Medium (ABM) supplemented with glucose as the sole carbon source, an easily degradable substrate (as detailed in Section 2.3). From the first re-cultivation of mixed cultures, two co-substrates, *p*-coumaric acid and glucose, were used. This approach aimed to stimulate bacterial growth, while allowing the microbial communities to acclimate to a phenolic environment, which resembles the conditions required for lignin degradation—the goal of this study.

Mixed cultures were inoculated onto solid medium (agar-solidified ABM with the same substrates), while oxygen-limited conditions were achieved by purging with Ar prior to sealing (see Section 2.5). This step was intended to enable the isolation of morphologically distinct colonies, allowing the degradation capacity of individual strains within each mixed community to be examined separately, independent of possible synergistic or antagonistic interactions within the consortium.

The isolates (for their isolation and liquid re-cultivation see Section 2.6) were tested for their ability to degrade *p*-coumaric acid as the sole carbon substrate (see Section 2.7). Their ability to grow in the presence of this substrate indicated that these strains could break down a simpler phenolic compound, suggesting their potential to degrade lignin as well. After this initial phase, both mixed and isolated cultures were re-cultivated in liquid medium with *p*-coumaric acid as the sole carbon substrate (see Section 2.4), which also provided insight into optimal re-cultivation intervals.

In the next phase, kraft lignin was added as a second substrate to both isolates and mixed cultures, alongside *p*-coumaric acid, to acclimate the microorganisms to the presence of actual lignin-like compounds (see Section 2.4). The isolates were then further evaluated for their ability to degrade kraft lignin alone and for their laccase and lignin peroxidase activities (as detailed in Section 2.8 and Section 2.9, respectively). The enzymatic activities of these enzymes were measured as indicative of oxidative lignin degradation pathways, which typically require oxygen or reactive oxygen species, such as hydrogen peroxide. The detection of such activities suggests that the isolates were not strictly anaerobic, but rather included microorganisms capable of functioning under oxygen-limited conditions, as facultative or microaerophilic strains.

Finally, a biomethane potential (BMP) test was conducted to assess the degradation performance of the mixed cultures on a natural lignocellulosic substrate (corn silage) (see Section 2.10). Mixed cultures were selected for this step because microbial consortia are more likely to enhance biogas production through bioaugmentation [20].

### 2.2. Sample Collection and Origin

Six soil samples were collected in October of 2022 from a wooded area near the University of Patras, Greece. Samples were taken either from the surface or by digging approximately 10 cm deep, targeting areas rich in decaying leaves and tree roots, where natural lignocellulose-degrading microbial communities are typically established. Although these surface soils are primarily aerated, they host indigenous tolerant anaerobic populations that remain viable in a dormant state or within anaerobic microsites [21,22,23]. In addition, two organic waste sources were used as potential inocula: two-phase olive mill wastewater (OMW) stored at room temperature for approximately one year, and anaerobic sludge derived from an experimental UASB reactor treating three-phase olive mill wastewater. The sampling sources are summarized in Table 1.

### 2.3. Preparation of Initial Suspensions and Initial Liquid Cultures

A total of 1 g of each sample was diluted in 100 mL of isotonic NaCl solution at a concentration of 9 g L^−1^ and stirred for 1 h. Subsequently, 5 mL of each prepared suspension was added into 50 mL of ABM, prepared according to Angelidaki et al. (2009) [24]. ABM contained L-cysteine hydrochloride (0.5 g L^−1^) as a reducing agent and resazurin (0.5 mg L^−1^) as a redox indicator. Cultures, with a working volume of 50 mL were incubated in sealed Duran bottles of 100 mL, as all the liquid cultures in this study. To ensure anaerobic conditions, prior to sealing, the vessels were reduced with Na_2_S · 9H_2_O to a final concentration of 0.025% and purged with argon gas (Ar) to displace any residual atmospheric oxygen from headspace. For the initial cultivation, *p*-coumaric acid and glucose were used at a concentration of 0.5 g L^−1^, each. The cultures were incubated at 37 °C for 7 days, with constant agitation.

### 2.4. Liquid Sub-Cultures

Subsequent re-cultures were performed every 7 days. For the first 5 re-cultures *p*-coumaric acid was used as sole substrate at a concentration of 0.5 g L^−1^. From that point onward, alkali kraft lignin was incorporated into ABM, as a second substrate, at a concentration of 0.5 g L^−1^. Due to the water-soluble nature of alkali kraft lignin and the buffering capacity of NaHCO_3_ system, stabilized with N_2_-CO_2_ (80:20 *v*/*v*) sparging of ABM, no additional treatment was required. Each re-culture was inoculated with 1 mL from the previous culture.

### 2.5. Preparation of Solid Cultures

Solid cultures were prepared using ABM supplemented with *p*-coumaric acid and glucose, each at a final concentration of 0.5 g L^−1^ and agar at 15 g L^−1^. The mixture was sterilized in 121 °C for 20 min and then allowed to partially cool down. Presterilized Petri dishes were filled with 15 mL of the prepared medium each.

After reaching room temperature, 1 mL of each liquid mixed culture was placed with pipette onto the dish and spread with a flame-sterilized glass rod, following the parallel streaking technique. Before sealing, each Petri dish was flushed with Ar to minimize oxygen presence. Incubation was carried out at 37 °C. For each of the 8 samples, 2 replicate plates were prepared, along with 2 uninoculated blanks serving as controls.

### 2.6. Isolation of Bacterial Colonies and Preparation of Their Liquid Cultures

After 10 days of incubation, microbial colonies displaying distinct morphological characteristics were observed. Under sterile conditions, individual colonies were excised by cutting the underlying solid medium and transferred into sterile Eppendorf tubes. The tubes were incubated at 37 °C prior to their transfer into liquid cultures.

Each isolated colony (1p–12p) was transferred into liquid anaerobic culture. For the initial cultivation of isolates, ABM was supplemented only with *p*-coumaric acid at a concentration of 0.5 g L^−1^. From the second cultivation onward, kraft lignin was added as a second substrate at the same concentration. Incubation was carried out at 37 °C under anaerobic conditions and sub-culturing was performed every 7 days.

### 2.7. Kinetics of p-Coumaric Acid Degradation

The aim of this experiment was the evaluation of the kinetics of *p*-coumaric acid degradation by isolates (1p–12p). As *p*-coumaric is one of the main products of lignin’s enzymatic degradation, isolates with the ability to degrade it are more probable candidates to show ligninolytic ability.

The liquid cultures of isolates were sub-cultured in ABM with *p*-coumaric acid as the sole substrate at a concentration of 0.5 g L^−1^. The inoculum for each culture was derived from the previous batch cultivation (which contained *p*-coumaric acid and glucose as substrates). To prevent the transfer of any residual glucose into the new cultures, 1 mL of inoculum was taken on the 7th day of incubation, when glucose was expected to be fully consumed.

Cultures, with a working volume of 50 mL were incubated in sealed Duran bottles of 100 mL under anaerobic conditions, in duplicate for each isolate and corresponding blanks (containing only ABM and the substrate). Incubation was performed at 37 °C and the experiment lasted for 5 days.

Biomass growth was assessed by measuring the optical density (OD) at 550 nm [25], while substrate degradation was monitored by measuring absorbance at 280 nm, the wavelength at which phenolic compounds, such as *p*-coumaric acid, exhibit maximum light absorption [26]. A Cary 50 UV–Vis spectrophotometer (Varian Inc., Palo Alto, CA, USA) was used, equipped with a glass and a quartz cuvette for the measurements at 550 nm and 280 nm, respectively. The photometric accuracy of the instrument is ±0.003 Abs units.

A standard curve for each isolate was established to correlate optical density (OD) with biomass concentration using the Total Suspended Solids (TSS) method, according to the Standard Methods for the Examination of Water and Wastewater [27], along with measurement of optical density at 550 nm. For this purpose, after measuring OD_550_, a known volume of culture sample (*V_s_*) was filtered under vacuum through a pre-dried and pre-weighted (*W_b_*) glass fiber filter (Whatman GF/F, pore size 0.7 μm). The filter was then rinsed with deionized water and dried at 105 °C until constant weight (*W_a_*). The increase in filter weight represented the dry biomass and the biomass concentration was calculated using the following equation:

(1) $$Biomass\;concentration\;\left[mg\;L^{-1}\right]=\frac{W_{a}-W_{b}}{V_{s}}$$

A standard curve for the conversion of absorption at 280 nm to the concentration of *p*-coumaric acid was acquired by preparing a solution of *p*-coumaric acid in deionized water at a concentration of 1 g L^−1^ and making subsequent dilutions to measure the absorbance at lower concentrations.

### 2.8. Biomass Growth with Kraft Lignin

Isolates (1p–12p) were cultivated using kraft lignin as the sole substrate in order to evaluate their biomass growth potential in lignin. The experiment was performed in duplicates of 50 mL volume each, with kraft lignin at a concentration of 0.5 g L^−1^ in ABM. 1 mL of each isolate was used as inoculum, previously cultivated in ABM containing *p*-coumaric acid and kraft lignin, and was collected on the 7th day of incubation to ensure that the residual concentrations of the substrates had been reduced, if not fully depleted. Incubation was carried out at 37 °C under anaerobic conditions, and the experiment lasted for 11 days.

It was observed that the color of the liquid cultures produced by kraft lignin changed over time, especially among different isolates. Therefore, the optical density (OD) at 550 nm (OD_550_) was measured before and after filtration using syringe nylon filters (Whatman, 0.22 μm pore size, 25 mm diameter). These measurements were used to correct the growth-related optical density for the background absorbance.

### 2.9. Evaluation of Enzymatic Activity

Monocultures were cultivated in 50 mL ABM supplemented with kraft lignin (0.5 g L^−1^). The inoculum for each monoculture was derived from the same isolate previously cultivated in ABM containing *p*-coumaric acid and kraft lignin in part of the acclimatization process. Enzymatic activity was evaluated during growth on kraft lignin rather than *p*-coumaric acid, as kraft lignin represents a more structurally complex and relevant to natural lignin substrate, while *p*-coumaric acid served as an initial screening compound.

Laccase enzymatic activity was measured according to Assavanig et al. (1992) [28]. After filtration of the culture samples through Whatman syringe nylon filters (0.22 μm pore size), 1 mL of the filtrate was mixed with 2 mL of 0.05 M citrate buffer (pH 3.1) containing *o*-dianisidine HCl at a concentration of 250 μg mL^−1^ in the buffer solution (3 mL reaction mixture in total). Optical density was measured at 450 nm using a 1 cm path length glass cuvette at room temperature (Cary 50 UV-Vis, Varian Inc., Palo Alto, CA, USA), while 2 mL of buffer solution with the addition of 1 mL of deionized water served as blank. One unit (U) of laccase activity was defined as the amount of enzyme causing an increase of 0.1 in absorbance per minute under the assay conditions. Enzyme activity was expressed as U mL^−1^ of culture filtrate.

Lignin peroxidase activity was determined based on the oxidation of veratryl alcohol to veratraldehyde [29]. The reaction mixture (total volume 2.5 mL) consisted of 1 mL of sodium tartrate buffer solution with a concentration of 125 mM and pH 3.0, 500 μL veratryl alcohol of 10 mM, 500 μL H_2_O_2_ of 2 mM solution and 0.5 mL of culture filtrate (Whatman syringe filters, 0.22 μm pore size). The reaction was initiated by the addition of hydrogen peroxide, which was defined as time zero, and the increase in absorbance was monitored at 310 nm using a 1 cm path length quartz cuvette (Cary 50 UV-Vis, Varian Inc., Palo Alto, CA, USA) at room temperature. A reaction mixture containing all reagents except culture filtrate, which was replaced by 0.5 mL of deionized water, was used as blank. One unit (U) of lignin peroxidase activity was defined as the amount of enzyme causing an increase of 0.1 in absorbance per minute at 310 nm under the assay conditions.

### 2.10. Biomethane Potential Experiment (BMP)

For the BMP assessment, corn silage was used as substrate, with volatile solids (VS) content of 394 g kg^−1^. Anaerobic sludge obtained from a 10 L laboratory-scale batch reactor treating agro-industrial wastewater was used as the primary inoculum, with VS concentration of 40 g L^−1^.

BMP assays were carried out according to the protocol of Angelidaki et al. (2009) using 160 mL serum bottles with a final working volume of 100 mL [24]. A constant volume of 10 mL anaerobic sludge was added as the base inoculum, and the amount of substrate was calculated at 0.25 g, to satisfy the requirement of the inoculum VS to substrate VS ratio to be equal to 4:1 [30]. Also, 20 mL of each mixed culture inoculum was added, and the remaining working volume was filled with ABM, which contained trace minerals, nutrients and vitamins.

All vials were flushed with a gas mixture of N_2_-CO_2_ (80:20 *v*/*v*) for 5 min to ensure anaerobic conditions and neutral pH and sealed immediately with butyl rubber septa and aluminum crimp caps. The bottles were incubated in an orbital shaking water bath at 37 °C with a rotation speed of 90 rpm.

All BMP tests were carried out in duplicate. In addition to the bioaugmented samples, two controls were included: (i) a base case consisting of corn silage and anaerobic sludge to show the potential of anaerobic sludge only, and (ii) a positive control containing cellulose as the sole substrate, used to verify the activity of the sludge inoculum.

Exhaustion of the samples was conducted daily to quantify the biogas production. The composition of the biogas, specifically CH_4_ and CO_2_ content, was determined using a gas chromatograph (Agilent Technologies 7890A, Agilent, Santa Clara, CA, USA) equipped with a thermal conductivity detector (TCD) and an HP-PLOT/Q capillary column. Gas samples of 0.3 mL were manually injected using a gas-tight microsyringe. All gas volumes were corrected to dry conditions and normalized to Standard Temperature and Pressure (STP) [31].

### 2.11. Modified Gompertz Model Fitting

The modified Gompertz model (Equation (2)) is frequently used to describe the cumulative biogas production profile during batch anaerobic digestion experiments, assuming a correlation with the microbial growth curve [32]. It is particularly suitable for representing the kinetics of the process when potential inhibitory effects influence microbial activity, which in our case may arise from substrate composition, accumulation of intermediates, or nutrient imbalances.

The model allows the estimation of kinetic parameters of maximum methane yield (*P_max_*, NmL g^−1^VS), the lag phase duration (*λ*, d), and the maximum methane production rate (*R_max_*, NmL g^−1^VS d^−1^). In the following equation *P* is the cumulative methane output (NmL g^−1^VS) and *t* is the experimental time (d).

(2) $$P=P_{max}\times \exp\left(-\exp\left(\frac{R_{max}\exp\left(1\right)}{P_{max}}\left(\lambda -t\right)+1\right)\right)\;\;$$

To evaluate the accuracy of the fitted model the coefficient of determination (*R*^2^) was calculated by Equation (3), where $y_{i}$ is the measured value, $\hat{y_{i}}$ is the predicted value, $\bar{y_{i}}$ is the mean of measured values and n is the number of predicted values. It is a useful statistical measure that represents the proportion of variance for the dependent variable that is explained by the independent variables in a regression model.

(3) $$R^{2}=1-\frac{\sum_{i=1}^{n}{\left(y_{i}-\hat{y_{i}}\right)}^{2})}{\sum_{i=1}^{n}{\left(y_{i}-\bar{y_{i}}\right)}^{2}}$$

### 2.12. Statistical Analysis

Statistical analysis of the main results was conducted using analysis of variance (ANOVA) in Minitab19 (Minitab LLC, State College, PA, USA). Differences among mean values were evaluated using the Tukey test, with significance level set at *p* < 0.05. The null hypothesis assumed no significant difference among group means; differences were considered statistically significant when the calculated *p*-value was below the defined threshold.

## 3. Results and Discussion

### 3.1. Isolation of Different Species

Following the procedure described in Section 2.1 using *p*-coumaric acid and glucose as co-substrates for growth, 8 mixed cultures were obtained. A total of 12 morphologically distinct colonies were then isolated from these 8 mixed cultures using the agar-solidified ABM (Table 2). No visible microbial growth was detected in the samples derived from *Quercus* sp. and *Pinus halepensis*.

### 3.2. p-Coumaric Kinetics

**Figure 2:**
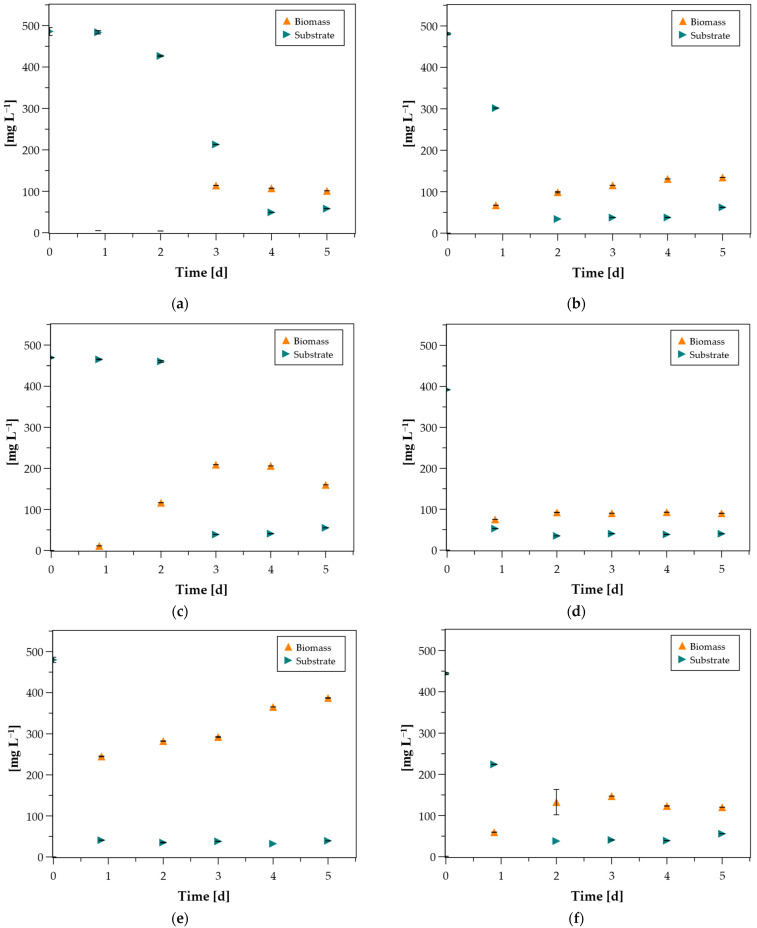

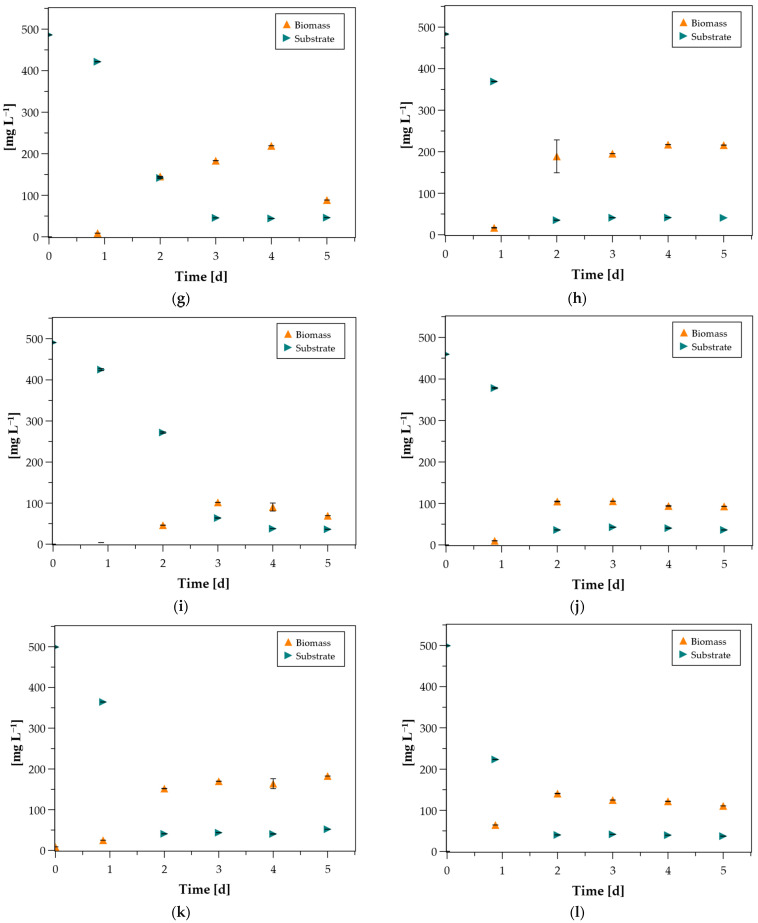
Concentration of biomass and substrate (*p*-coumaric acid) of each pure culture. (**a**) 1p, (**b**) 2p, (**c**) 3p, (**d**) 4p, (**e**) 5p, (**f**) 6p, (**g**) 7p, (**h**) 8p, (**i**) 9p, (**j**) 10p, (**k**) 11p, (**l**) 12p. Data are the mean values ± SD (n = 3).

The evolution of the culture growth and the *p*-coumaric degradation for each one of the 12 isolates is presented in Figure 2. The residual OD_280_ observed in all diagrams is attributed to background contributions of the medium and other non-specific components. This conclusion is supported by the fact that the residual absorbance remained nearly identical across most of the samples. However, a distinct trend was observed for isolates 2p, 3p, and 6p, and to a lesser extent for 1p and 11p. In these cases, a slight increase in absorbance at 280 nm was detected around day 5. This transient increase may suggest the accumulation of intermediate compounds, such as 4-hydroxybenzoic acid, a phenolic intermediate of *p*-coumaric degradation [33].

All isolates managed to grow in *p*-coumaric acid and degrade 100% of the initial *p*-coumaric acid within 2 to 4 days in most cases. The maximum biomass concentration was observed in isolate 5p, reaching 387 mg L^−1^. Isolates 3p, 7p and 8p followed with maximum biomass concentrations 209 mg L^−1^, 219 mg L^−1^ and 217 mg L^−1^, respectively. Most of the other isolates exhibited peak biomass between 100 and 200 mg L^−1^, with the lowest biomass concentration observed for isolate 4p which did not exceed 100 mg L^−1^. The majority of isolates reached their maximum biomass accumulation between days 2 and 4, except from 2p, 5p, 8p and 11p which reached their maximum on day 5, indicating that growth was still ongoing. For example, *Sphingobacterium* sp. HY-H reached OD_600_ ≈ 1.0 within two days when grown aerobically on *p*-coumaric acid at 150 mg L^−1^ [17], while Trautwein et al. (2012) showed that the anaerobic isolate *Aromatoleum aromaticum* achieved a maximum OD_660_ of 0.35 over 40 h when cultivated at an initial concentration of 328 mg L^−1^ of *p*-coumaric acid [34].

In all cases, with the exception of isolates 1p, 9p and 3p, a clear correlation was observed between the onset of substrate consumption and the initiation of biomass growth, indicating that *p*-coumaric acid was directly utilized as a carbon source. In contrast, isolate 3p exhibited measurable growth during the first two days without substantial apparent degradation of the substrate. Since *p*-coumaric acid consumption was monitored spectrophotometrically at 280 nm, it is possible that isolate 3p partially transformed *p*-coumaric acid into phenolic intermediates that still absorb strongly at this wavelength. These intermediate compounds may have supported initial microbial growth while simultaneously contributing to the absorbance signal at 280 nm, thereby masking the actual extent of substrate conversion during the early stages of cultivation [33]. On the other hand, a contrasting observation was made for isolates 1p and 9p. While a steady decrease in the substrate concentration was recorded from the early stages, these isolates exhibited a lag phase of 1 to 2 days in their biomass growth. This decoupling of substrate utilization and cell proliferation can be attributed to the high metabolic cost of enzyme secretion [35].

Highest substrate removal rate (438 mg L^−1^ d^−1^) was recorded for isolate 5p, which completely degraded *p*-coumaric acid within the first day of cultivation, in contrast with isolate 1p with the slowest consumption rate and total removal observed on the 4th day. A strain of *Azotobacter* sp. had also shown the ability to degrade *p*-coumaric acid when it was cultivated in 30 °C with 50% of the substrate consumed by the 5th day [36]. These results are aligned with other studies reporting variable degradation efficiencies for aromatic compounds. *Pseudomonas* sp., for instance, demonstrated rapid substrate use within 9 h at even higher *p*-coumaric initial concentrations [37]. Finally, a death phase was identified for the majority of the isolates after the significant decrease in substrate. This decline in biomass was most pronounced for isolates 3p and 7p, while a lower death rate was observed for isolates 1p, 6p, 9p, 10p, and 12p.

### 3.3. Growth of Isolates in Kraft Lignin

**Figure 3:**
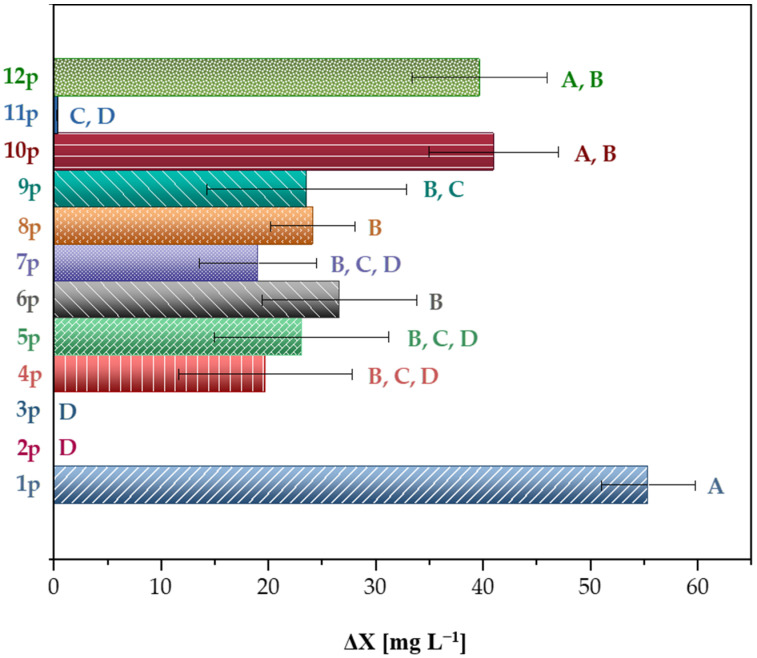
Total biomass growth (ΔΧ, mg L^−1^) in kraft lignin for each of the 11 isolates until the end of an 11-day experiment. Data are the mean values ± SD (n = 3). Data that do not share a common letter (A, B, C, D) are statistically different based on Tukey test (*p* < 0.05).

In Figure 3 the net increase in biomass is shown for each isolate cultivated in kraft lignin as sole substrate. The highest biomass increase was observed for isolate 1p at 55 mg L^−1^, followed by isolates 10p and 12p at 41 and 40 mg L^−1^, respectively, whereas isolates 2p and 3p shown negligible ability for growth.

For the cultures that demonstrated measurable biomass formation, the increase in optical density ranged from 10^−3^ for isolate 11p to 10^−2^ for the remaining isolates. Although these values appear low, they are consistent with the limited growth typically observed when lignin serves as the sole carbon source under anaerobic conditions. The anaerobic bacterium *Tulomonas lygnolytica* cultivated in lignin substrate exhibited an increase in optical density of 0.11 abs measured at 600 nm [38]. Similarly, bacterial strains such as *Microbacterium phyllosphaerae* and *Ochrobactrum* sp. in a similar experiment presented an increase in optical density of about 0.005 abs (at 430 nm) [39].

### 3.4. Enzymatic Activity

#### 3.4.1. Lignin Peroxidase Evaluation

**Figure 4:**
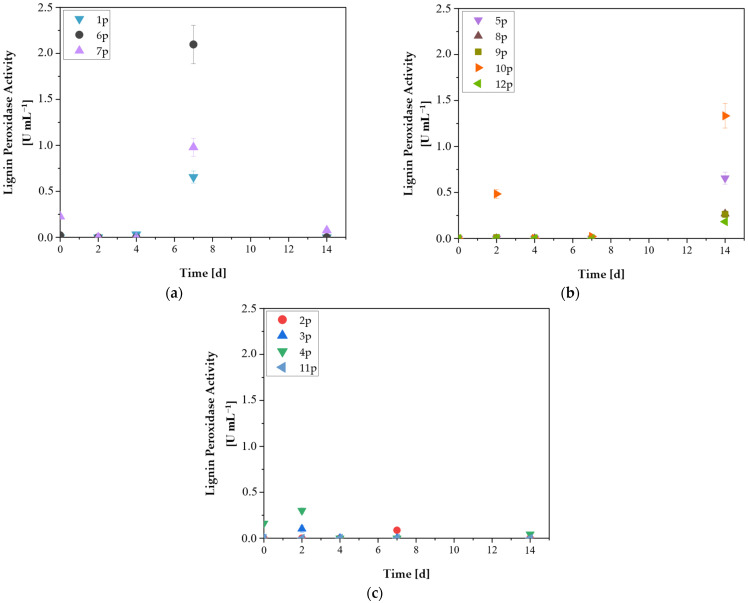
Volumetric enzymatic activity of Lignin Peroxidase (LiP) during the cultivation period of the isolates 1p–12p. (**a**) Isolates exhibiting highest volumetric LiP activity on the 7th day, (**b**) Isolates exhibiting highest volumetric LiP activity on the 14th day, (**c**) Isolates exhibiting the lowest volumetric LiP activity. Data are the mean values ± SD (n = 3).

The Lignin Peroxidase (LiP) volumetric activity of isolates is presented in Figure 4a–c. Isolates 6p, 7p and 1p exhibited their highest LiP on the 7th day of cultivation, reaching 2.10 U mL^−1^, 0.98 U mL^−1^ and 0.66 U, mL^−1^, respectively (Figure 4a). Mei et al. (2020) reported that *Bacillus amyloliqufaciens* cultivated on tobacco residue and lignin substrates exhibited its maximum LiP activity on 6th day, reaching 0.42 U mL^−1^ [40]. Higher LiP production has also been documented in the work of Batayyib et al. (2022), where soil-derived *Actinomycetes* and *Bacillus* spp. produced 3.8 and 2.4 U mL^−1^, respectively, under aerobic although conditions [41].

As shown in Figure 4b, isolates 5p, 8p, 9p, 10p and 12p reached their maximum LiP activity on day 14 of incubation, with isolate 10p exhibiting the highest value at 1.33 U mL^−1^. This delayed peak is rather unusual for ligninolytic bacteria, as they typically reach maximum LiP activity earlier in their growth phase. Fungi, on the other hand, are more commonly associated with delayed LiP production. For example, *Trametes trogii*, a white-rot fungus, achieved its maximum LiP activity between 15 and 20 days, reaching 0.18 U mL^−1^ [42].

In Figure 4c, isolates with the lowest LiP volumetric activity are presented. Isolate 11p exhibited no measurable response in the period of 14 days. Isolates 3p and 4p demonstrated a slight increase on day 2, reaching 0.1 and 0.3 U mL^−1^, respectively. This short-term pattern has also been reported by Lai et al. (2017) [43], who found that several bacterial isolates obtained from guaiacol-enriched solid cultures produced maximum LiP activity within 24–48 h. Finally, isolate 2p showed a peak on day 7, although its volumetric activity remained an order of magnitude lower than the isolates of Figure 4a,b.

Overall, isolates 1p (0.66 U mL^−1^), 5p (0.66 U mL^−1^), 6p (2.1 U mL^−1^), 7p (0.98 U mL^−1^) and 10p (1.33 U mL^−1^) demonstrated volumetric LiP activities comparable to those reported for aerobic bacteria. These findings are noteworthy because the cultures were maintained in sealed liquid media where oxygen availability was intentionally minimized, although strictly anaerobic conditions could not be guaranteed via, e.g., continuous N_2_ sparging. The detection of LiP activity under these low-oxygen conditions suggests that these isolates may include facultative anaerobes or microaerophiles capable of expressing oxygen-dependent ligninolytic enzymes. Conversely, isolates with negligible LiP activity, particularly 11p (0.0 U mL^−1^), 2p (0.09 U mL^−1^), 3p (0.10 U mL^−1^) and 12p (0.18 U mL^−1^), are more consistent with strictly anaerobic metabolisms.

#### 3.4.2. Laccase Evaluation

**Figure 5:**
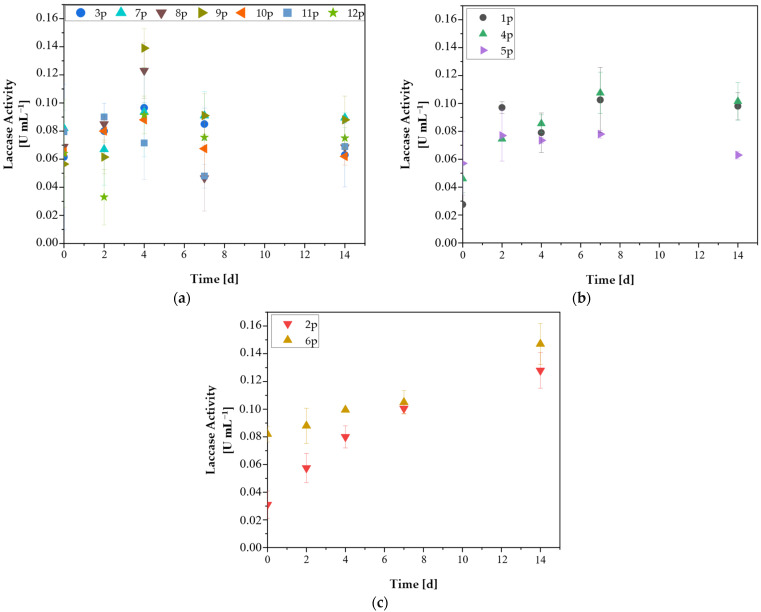
Laccase enzymatic activity [U mL^−1^] of isolates 1p–12p during a 14-day experiment. (**a**) Isolates exhibiting highest volumetric Lac activity on the 2nd and 4th day, (**b**) Isolates exhibiting highest volumetric Lac activity on the 7th day, (**c**) Isolates exhibiting continuously increasing volumetric Lac activity. Data are the mean values ± SD (n = 3).

Laccase (Lac) activity of the isolates is presented in Figure 5. Laccase production was observed in all isolates from the first day of cultivation and gradually increased until the 4th and 7th day, when most samples exhibited their maximum values.

Specifically, Figure 5a shows the isolates that exhibited maximum volumetric laccase activity on the 2nd and 4th days of cultivation. Particularly, isolate 9p exhibited 0.14 U mL^−1^ highest laccase activity, while 8p resulted in its highest 0.12 U mL^−1^. Both isolates showed moderate biomass growth (approximately 25 mg L^−1^). Interestingly, while isolates 3p, 7p, 10p, and 11p exhibited similar maximum enzymatic activities of nearly 0.10 U mL^−1^, 3p and 11p showed negligible biomass growth. This discrepancy suggests a metabolic shift toward self-preservation and maintenance rather than cellular proliferation, where energy is prioritized for the synthesis of extracellular enzymes to modify the recalcitrant environment [35].

A second group of isolates (4p, 1p, and 5p) exhibited maximum laccase activity (0.11, 0.10, and 0.08 U mL^−1^, respectively) on day 7 (Figure 5b). Similarly, under aerobic conditions, *Lysinibacillus* sp., *Acinetobacter* sp., and *Bacillus* sp. grown on kraft lignin exhibited early laccase production, typically beginning between days 2 and 3, with maximum values observed between days 4 and 6. Notably, that study found that lignin peroxidase activity was about 3.5 times higher than laccase activity, suggesting that laccase plays a more auxiliary role in these bacterial ligninolytic systems [44].

Figure 5c shows isolates with continuously increasing laccase activity up to day 14. This includes isolate 6p, which reached a maximum of 0.15 U mL^−1^, and 2p, which reached a maximum of 0.13 U mL^−1^. This gradual trend resembles findings from *Streptomyces cyaneus*, which exhibited a consistent rise in laccase activity up to day 20 when cultivated on various lignocellulosic substrates, such as soy, wood, and sawdust, reaching a maximum of 0.06 U mL^−1^ [45]. *Mycobacterium smegmatis* was also shown to degrade up to 50% of kraft lignin within 8 days, reaching 0.18 U mL^−1^ Lac activity [46], a value comparable to the highest results in the present study. Although isolate 2p demonstrated laccase production capability, it exhibited negligible biomass growth, similarly to isolates 3p and 11p. According to Sajjad et al. (2025) soil bacteria particularly *Actinomycetes*, are capable of solubilizing lignin, although their ability to extensively metabolize it is lower compared to fungi [47]. Moreover, in both samples 2p and 6p, the continuous enzyme production observed until day 14 may indicate that lignin depolymerization was still ongoing during this period.

The presence of laccases in these experiments, even at low levels, may again indicate microaerophilic conditions. However, previous studies have demonstrated that laccase-like enzymes can be encoded by bacteria, including strictly anaerobic species such as *Geobacter metallireducens.* The heterologously expressed GeoLacc enzyme from this organism was shown to require oxygen as the terminal electron acceptor and retain activity under microaerophilic conditions. It is hypothesized that the enzyme functions as a protective oxidase against trace oxygen, thereby safeguarding the anaerobic bacterium [48].

### 3.5. BMP Evaluation

**Figure 6:**
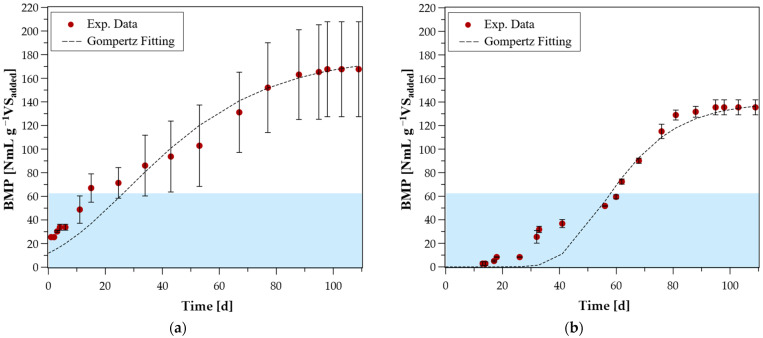

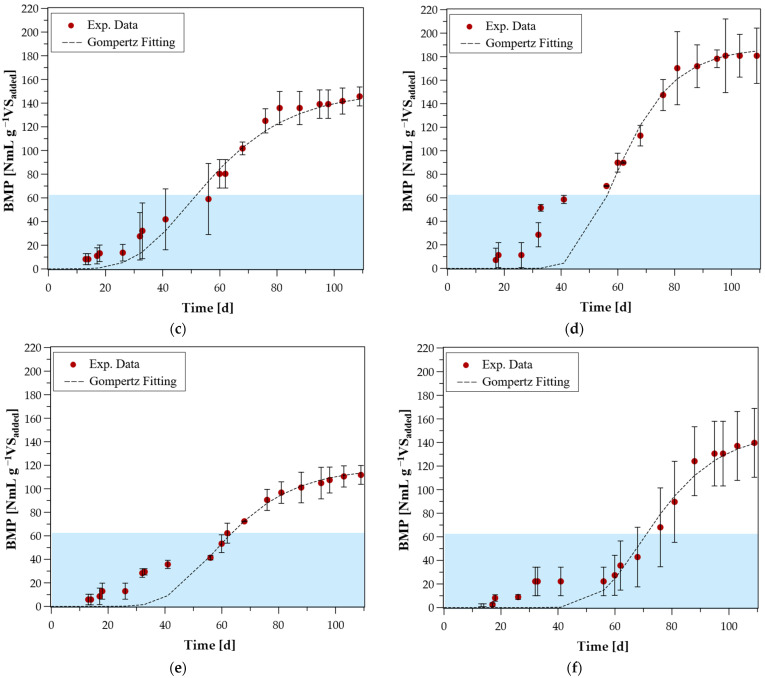

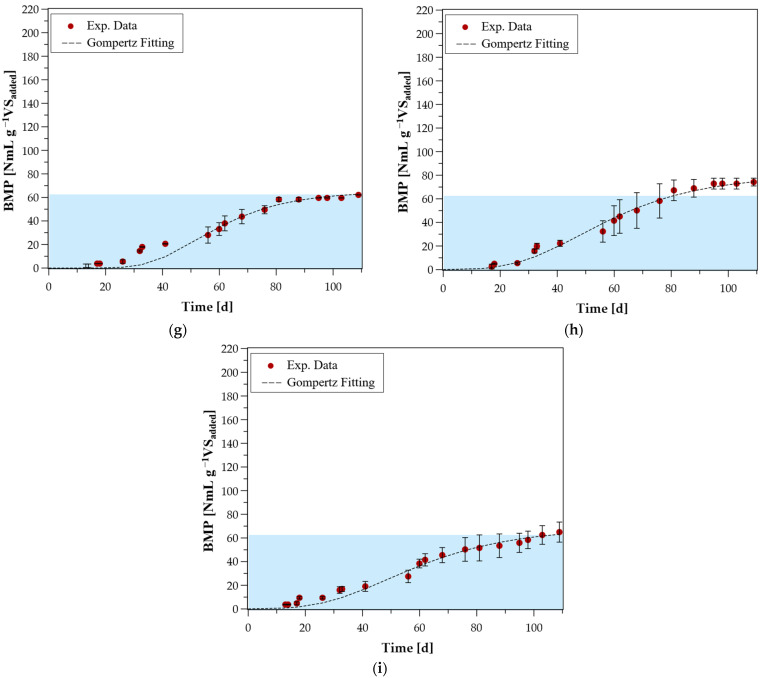
Cumulative methane production evolution [NmL CH_4_ g^−1^VS_added_] over experimental time [d]. (**a**) Anaerobic sludge from UASB, (**b**) Two-phase OMW, (**c**) *Cupressus sempervirense* ^b^, (**d**) *Pinus halepensis* ^a^, (**e**) *Pinus halepensis* ^b^, (**f**) *Olea europaea* ^a^, (**g**) *Eucalyptus globulus* ^a^, (**h**) *Quercus* sp. ^a^, (**i**) Control. The blue area represents the control level in all diagrams.

Figure 6 presents the results of the biomethane potential (BMP) experiment evaluating the degradation of corn silage following the addition of each enriched mixed culture (Table 1) to the anaerobic sludge inoculum as a bioaugmentation agent. Prior to their application in the BMP assays, the mixed cultures had undergone sequential recultivations with progressive acclimation to *p*-coumaric acid and subsequently to kraft lignin, as described in Section 2.4. The Gompertz model was fitted to all samples (see Table 3) because plateau phases were not clearly reached in some cases despite the 109-day duration of the experiment. Overall, the fitting accuracy was satisfying with R^2^ ranging from 0.92 to 0.99.

However, in some instances and especially in Figure 6b,d–f, an initial methane production was observed during the first days of cultivation which the monophasic Gompertz model could not fully incorporate. This discrepancy is attributed to the complex composition of corn silage, which consists of both readily biodegradable fractions (e.g., soluble sugars) and recalcitrant lignocellulosic components. In this case sigmoidal models prioritize the main growth phase, often resulting in a predicted lag phase that overlooks early-stage hydrolysis in heterogeneous substrates [49].

The control sample (5i) reached 65 NmL CH_4_ g^−1^VS_added_ and is marked as a blue shading area in the graphs. With the exception of samples from *Eucalyptus globulus* ^a^ and *Quercus* sp. ^a^, all the samples exhibited significant bioaugmentation ability. The sample inoculated with a mixed culture derived from *Pinus halepensis* ^a^ exhibited the highest methane accumulation, reaching 181 NmL g^−1^VS.

Most samples exhibited a lag phase ranging from 25 days (as observed in the control) to 40 days. Notable deviations were observed in the sample inoculated with a culture derived from anaerobic sludge treated in a UASB reactor, which exhibited methane production from day 1, and in the *Olea europaea* ^a^ sample, which exhibited an extended lag phase of over 50 days.

The bioaugmentation percentage is calculated based on the maximum methane potential estimated from the fitted Gompertz model to eliminate misinterpretations arising from samples that did not reach a stable plateau. The best bioaugmentation was observed in the sample with *Pinus halepensis* ^a^, yielding a 174% increase compared to the control.

Samples bioaugmented with mixed cultures from two-phase OMW, *Cupressus semprevirense* ^b^, anaerobic sludge from UASB, and *Olea europaea* ^a^ resulted in bioaugmentation ranging from 104% to 163%. In contrast, *Pinus halepensis* ^b^ followed with double accumulation. *Quercus* sp. ^a^ only resulted in modest bioaugmentation of 17%.

Compared to previous studies, Weiß et al. (2016) [50] improved the anaerobic digestion of lignocellulosic substrates (maple leaves and straw) by using a mixed culture of hydrolytic bacteria isolated from anaerobic sludge from biowaste treatment. They reported a 42% higher methane yield than the non-augmented control. A similar enhancement (36%) was achieved using a mixed culture from a goat’s rumen, a natural cellulolytic environment, for the anaerobic digestion of wheat straw [51]. Also, Nurika et al. (2022) [52] reported up to a 920% increase in biomethane production when oil palm empty fruit bunches were pretreated individually with bacterial cultures of *Comamonas testosteroni*, *Agrobacterium* sp., *Lysinibacillus sphaericus*, and *Paenibacillus* sp. Each strain was applied separately and achieved lignin degradation efficiencies ranging from 12.3 to 25.8%, thereby enhancing substrate accessibility and improving subsequent methane yield [52].

### 3.6. Final Evaluation and Bacterial Selection

The final conclusion and selection of the most promising bacterial isolates for lignin biodegradation were based on a stepwise decision-making algorithm, as summarized in Table 4.

Initially, all isolates fulfilled the first criterion because the results of the *p*-coumaric acid degradation experiment were positive for all isolates. The second criterion was based on the ability of each isolate to degrade kraft lignin. According to the experimental results (Figure 3), the isolates grouped under clusters ‘A’, ‘A, B’, and ‘B’ exhibited the highest growth on kraft lignin. These included isolates 1p, 6p, 8p, 10p, and 12p. The third criterion was based on the ability of the mixed cultures to enhance methane production in the BMP assays. Cultures that produced higher volume of methane than the control were considered successful. Based solely on this third criterion, all mixed cultures would have been accepted, except for *Eucalyptus*, which produced less methane than the control. Isolates 8p, 9p, and 10p, which originated from *Eucalyptus*, were thus excluded because they did not satisfy this requirement. Ultimately, isolates 1p, 6p, and 12p were the only ones that met all three selection criteria and were deemed the most promising for lignin degradation.

The results of the enzymatic activity of LiP and Lac further supported the selection process. Even under conditions of limited oxygen, most of the isolates exhibited significant enzymatic activities. Isolate 2p, which did not grow on lignin substrate, expressed only Lac activity, peaking on day 14 (0.13 U mL^−1^), i.e., after the 11-day growth period on lignin had ended. Similarly, isolate 3p, which also failed to grow on lignin, showed no LiP activity and only Lac activity, peaking at 0.10 U mL^−1^ on day 4. Since laccases are considered less effective than peroxidases in lignin breakdown, their exclusive presence may explain why these isolates cannot utilize lignin.

In contrast, isolate 1p, which exhibited the greatest growth on kraft lignin, displayed both LiP and Lac activity. The LiP peak occurred on day 7 (0.66 U mL^−1^), while the Lac activity began as early as day 1 and remained consistently high until the end of the experiment (0.10 U mL^−1^). This enzymatic “cocktail” could explain the robust growth of isolate 1p in lignin.

Isolates 10p and 12p showed high biomass accumulation on lignin as well. Isolate 10p produced both enzymes. LiP peaked early (day 2, 0.50 U mL^−1^), while Lac maintained steady levels throughout (0.06–0.09 U mL^−1^). Isolates 11p and 12p expressed very low enzymatic activities according to both LiP and Lac (11p: LiP not detected; Lac: 0.09 U mL^−1^) and 12p: LiP: 0.18 U mL^−1^, Lac: 0.09 U mL^−1^). These results suggest that these isolates may be strictly anaerobes. The fact that these isolates are part of a mixed culture derived from *Pinus halepensis^a^*, which exhibited the highest bioaugmentation, further supports this hypothesis.

Finally, isolate 6p, which showed satisfactory growth on lignin, exhibited the highest LiP activity (2.10 U mL^−1^), while its Lac activity started at the highest level on day 1 and increased until the end of the experiment (0.15 U mL^−1^).

## 4. Conclusions

This study proposed and evaluated a strategy for isolating and assessing native microbial strains and communities from natural environments where lignocellulosic biomass undergoes degradation. The selection process involved a stepwise evaluation of simpler aromatic compounds, such as *p*-coumaric acid, followed by more recalcitrant substrates, such as kraft lignin, and ultimately a real lignocellulosic substrate: corn silage.

All of the isolated cultures demonstrated the ability to degrade *p*-coumaric acid. Isolate 5p achieved the highest biomass concentration (387 mg L^−1^), and isolates 4p and 5p demonstrated the fastest degradation, consuming all of the initial *p*-coumaric acid within the first day of cultivation. However, degrading *p*-coumaric acid does not necessarily guarantee the ability to break down lignin. Further acclimating *p*-coumaric acid isolates to harder substrates in steps may enhance their ability to break down lignin or, at least, increase their resistance.

Focusing on key enzymes involved in lignin degradation, such as LiP and Lac, an analysis of enzymatic activity provided valuable insights into the metabolic potential of selected isolates. For example, isolate 6p, one of five isolates that exhibited significant growth on kraft lignin, displayed also high LiP activity (2.10 U mL^−1^), supporting its ligninolytic capability even under conditions of limited oxygen.

Furthermore, mixed cultures incorporating these promising isolates resulted in significant bioaugmentation in most cases. The highest increase in methane yield (174%) was observed in the mixed culture derived from surface soil under *Pinus halepensis*. This culture contained the isolates 11p and 12p, which are the most likely to be anaerobes, indicating its strong potential for lignocellulose degradation under anaerobic conditions.

Future work could involve testing individual isolates within consortia separately to better understand microbial interactions, whether synergistic or antagonistic. More comprehensive enzymatic profiling, including additional ligninolytic enzymes (e.g., manganese peroxidase), could clarify the metabolic capabilities involved in lignin breakdown.

In conclusion, native bacterial isolates obtained from environments naturally rich in decaying lignocellulosic biomass are promising candidates for bioaugmentation strategies. Applying them could enhance the degradation of the recalcitrant component of lignin and contribute to the more efficient valorization of lignocellulosic waste into value-added products, such as biogas, in alignment with the principles of circular bioeconomy.

## Figures and Tables

**Table 1:** Sampling sources.

Sampling Sources	Coordinates of Sampling Points
*Pinus halepensis* ^a,b^	38.2866768° N, 21.7972988° E
* Cupressus sempervirens * ^b^	38.2856300° N, 21.7976297° E
*Quercus* sp. ^b^	38.2867566° N, 21.7971000° E
* Olea europaea * ^a^	38.2855413° N, 21.7981769° E
* Eucalyptus globulus * ^b^	38.2867118° N, 21.7975164° E
Two-phase OMW	
Sludge from UASB reactor	

^a^ Sample derived from surface soil; ^b^ sample derived from 10 cm below surface; ^a,b^ samples derived from both depths.

**Table 2:** Isolates derived and their corresponding mixed cultures.

Mixed Culture Source	Derived Isolates
Anaerobic sludge from UASB	1p
Two-phase OMW	2p, 3p
*Cupressus sempervirens* ^b^	4p, 5p, 7p
*Olea europaea* ^a^	6p
*Eucalyptus globulus* ^b^	8p, 9p, 10p
*Pinus halepensis* ^a^	11p, 12p

^a^ Sample derived from surface soil; ^b^ sample derived from 10 cm below surface.

**Table 3:** Estimated parameters of modified Gompertz model.

	* P_max_* [NmL CH_4_ g^−1^VS]		* R_max_* [NmL CH_4_ g^−1^VS d^−1^]		* λ * [d]		* R * ^ 2 ^
Anaerobic sludge from UASB	179.9	(154.7–205.1)	2.38	(1.77–2.99)	0.0	(−7.4–7.4)	0.95
Two-phase OMW	140.0	(119.5–160.5)	3.48	(1.86–5.10)	40.0	(30.1–49.9)	0.95
*Cupressus sempervirense* ^b^	150.0	(132.1–167.9)	2.86	(2.15–3.57)	30.0	(23.6–36.5)	0.97
*Pinus halepensis* ^a^	187.8	(156.3–219.4)	5.53	(1.91–9.14)	45.0	(33.6–56.4)	0.93
*Pinus halepensis* ^b^	117.8	(93.5–142.2)	2.68	(1.20–4.15)	40.0	(27.8–52.2)	0.92
*Olea europaea* ^a^	150.0	(117.8–182.2)	3.59	(2.35–4.83)	53.6	(47.7–59.6)	0.96
*Eucalyptus globulus* ^a^	65.0	(55.1–74.9)	1.40	(0.87–1.93)	35.0	(25.8–44.2)	0.95
*Quercus* sp. ^a^	80.0	(72.2–87.8)	1.27	(1.07–1.46)	25.0	(20.6–29.4)	0.99
Control	68.5	(58.8–78.2)	1.05	(0.83–1.27)	25.0	(18.8–31.2)	0.97

^a^ Sample derived from surface soil; ^b^ sample derived from 10 cm below surface.

**Table 4:** Summary of the reasoning process of the algorithm for selecting the optimal microbial culture for lignin biodegradation. Y (Yes)/N (No) indicates whether each sample meets each criterion or not (n.d. stands for “not detected”).

Mixed Cultures	Isolates	Growth in *p*-Coumaric Acid [Y/N]	Growth in Kraft Lignin [Y/N]	Bioaugmentation [%]	Fulfillment of 3 Criteria [Y/N]	Maximum LiP Activity [U mL^−1^]	Maximum Lac Activity [U mL^−1^]
Anaerobic sludge from UASB	1p	Y	Y	163	Y	0.66	0.10
Two-phase OMW	2p	Y	N	104	N	n.d.	0.13
	3p	Y	N			n.d.	0.10
*Cupressus sempervirense* ^b^	4p	Y	N	119	N	0.30	0.11
	5p	Y	N			0.66	0.08
	7p	Y	N			0.98	0.09
*Pinus halepensis* ^a^	11p	Y	N	174	N	n.d.	0.09
	12p	Y	Y			0.18	0.09
*Olea europaea* ^a^	6p	Y	Y	119	Y	2.10	0.15
*Eucalyptus globulus* ^a^	8p	Y	Y	n.d.	N	0.27	0.12
	9p	Y	N			0.26	0.14
	10p	Y	Y			1.33	0.09
*Pinus halepensis* ^b^	n.d.	-	-	72	-	-	-
*Quercus* sp. ^a^	n.d.	-	-	17	-	-	-

^a^ Sample derived from surface soil; ^b^ sample derived from 10 cm below surface.

## Data Availability

The original contributions presented in this study are included in the article. Further inquiries can be directed to the corresponding author.

## Abbreviations

The following abbreviations are used in this manuscript:

LB	Lignocellulosic biomass
H	*p*-hydroxyphenyl monolignol
G	Guaiacyl
S	Syringyl
LCC	Lignin carbohydrate complex
Lac	Laccase
MnP	Manganese peroxidase
LiP	Lignin peroxidase
VP	Versatile peroxidase
ABM	Anaerobic basic medium
OMW	Olive mill wastewater
UASB	Uplow anaerobic sludge banket reactor
Ar	Argon
OD	Optical density
TSS	Total suspended solids
U	Unit of enzymatic activity
VS	Volatile solids
TCD	Thermal conductivity detector
STP	Standard temperature and pressure
NmL	Normalized mL in STP conditions
SD	Standard deviation
AD	Anaerobic digestion
BMP	Biomethane potential
Y	Yes
N	No
n.d.	Not detected
